# Environmental detection of *Fasciola hepatica* by loop-mediated isothermal amplification

**DOI:** 10.7717/peerj.13778

**Published:** 2022-08-04

**Authors:** Lily Tran, Hayley Toet, Travis Beddoe

**Affiliations:** Department of Animal, Plant and Soil Sciences, School of Agriculture, Biomedicine and Environment, La Trobe University, Bundoora, Victoria, Australia

**Keywords:** *Fasciola hepatica*, LAMP, Environment, Fluke, Parasite, Field, Detection

## Abstract

*Fasciola hepatica*, commonly referred to as liver flukes, is a substantial zoonotic parasitic disease of humans and livestock globally. While infection is readily controlled by anthelmintics, namely triclabendazole, the heavy reliance on triclabendazole has resulted in drug resistance appearing worldwide. Due to drug resistance, it is imperative to adopt an integrated parasite management program to preserve the efficacy of currently available anthelmintics. A integrated liver fluke management plan would benefit from a simple rapid, field-deployable diagnostic for detection of *F. hepatica* in environment and the host. Therefore, a rapid DNA test using loop-mediated isothermal amplification was developed and optimised for the detection of *F. hepatica* from faecal and water samples to enable the detection of parasites both within the host and from the environment. The assay presented here is fast, with amplification in ≤20 min, and highly sensitive, with a detection limit of 5 × 10^−4^ ng/µL. The workflow presented here provides a time to result of ≤60 min without requiring a commercial kit for the extraction of DNA from faecal and water samples, and pending further validation from field-samples, could potentially be used to enable real-time decision making to mitigate parasite prevalence on a farming property and with no requirement for sample transportation.

## Introduction

*Fasciola hepatica* is a globally distributed, zoonotic trematode causing the disease Fascioliasis. Commonly referred to as liver flukes, these parasites are found in temperate regions such as Europe and Australia (Durr, Tait & Lawson, 2005). Infection is most common in livestock ruminants with an estimated 600 million animals infected worldwide (Wilson et al., 2011). Fascioliasis is therefore a substantial animal health issue costing the global agricultural sector USD $3.2 billion annually from a reduction in produce yields including meat, milk, and wool (Piedrafita et al., 2010; Walker et al., 2004). In Australia alone, Fascioliasis costs the agricultural sector AUD $50–80 million *per annum*, with a further AUD $10 million spent on flukicidal treatments to reduce disease burden (Lane et al., 2015).

The management of the disease is primarily by the administration of Triclabendazole (TCBZ), an anthelmintic first introduced in the 1980’s, used frequently for Fascioliasis control (Walker et al., 2004). To date, it is the only flukicide that is lethal to both newly excysted juveniles (NEJs); responsible for acute Fascioliasis and clinical symptoms, and adults; associated with chronic disease resulting in ongoing production losses and treatment costs (McManus & Dalton, 2006; Scarcella et al., 2012). Long-term alternatives, for example vaccines, are still being researched with varying levels of efficacy, hindering commercialisation (Toet, Piedrafita & Spithill, 2014). Consequently producers have limited options and must rely on flukicides for parasite control (Hanna et al., 2015). Sustained overuse and misuse of TCBZ has resulted in the emergence of TCBZ-resistant flukes (TCBZ-R) with cases reported in Europe, South America, and Australia, in both livestock and humans (Brockwell et al., 2014; Cabada et al., 2016; Gordon et al., 2012; Kelley et al., 2016; Winkelhagen et al., 2012). Combination flukicides are available, but are underutilized due to milk withholding periods in dairy cattle (Overend & Bowen, 1995).

The complex multi-host lifecycle of *F. hepatica* is essential for its development and survival. Mature flukes in the definitive host bile ducts produce eggs which are shed in faeces into the environment. These embryonate in water and miracidia emerge seeking out a snail intermediate host, commonly Lymnaeid snails. Within the snail host, *F. hepatica* undergoes multiple developmental stages before emerging as free-swimming cercariae encysting on nearby vegetation as metacercariae which livestock or humans ingest. They then excyst emerging as juveniles migrating through the liver parenchyma and mature after 6–8 weeks in the bile ducts, repeating the lifecycle (Young et al., 2010). Currently detection methods for *F. hepatica* are reliant on diagnosing the adults commonly copromicroscopy such as faecal egg counts, Kato-Katz thick smear, or FLOTAC methods (Zárate-Rendón et al., 2019). These methods are relatively simple and low-cost, and can detect current mature infections. However, they are time consuming and prone to errors due to low sensitivity and specificity in addition to intermittent egg shedding by *F. hepatica* (Brockwell et al., 2013; Mazeri et al., 2016).

Improvements have been made with the development of serological and coproantigen ELISAs for *F. hepatica* detection. However, these methods have some limitations, including: serum ELISAs returning potential false positive results from long-lived antibody titres persisting after infection has cleared; or coproantigen methods being limited to detecting patent infections (Brockwell et al., 2013; Mezo et al., 2004). These methods demonstrate high sensitivity but are still restricted to diagnosing established infections within the host from direct sampling through blood and/or faecal collection. Additionally, ELISA detection is high with the average cost of submitting a coproantigen ELISA (cELISA) sample estimated to be AUD $22/sample with testing restricted to a well-equipped central laboratory confirm infection (Agriculture Victoria, 2019; Elliott et al., 2015; Molloy et al., 2005). The requirement of host samples makes disruption of the fluke lifecycle difficult as these life stages reside within the definitive host. Though these are useful in influencing treatment decisions, these methods are unable to be applied to the aquatic stages of the parasite, which ultimately give rise to the infectious metacercariae. Shifting focus to these stages would enable indirect sampling to determine parasite prevalence in an environment without having to individually sample animals.

Improvements to *F. hepatica* detection have seen numerous nucleic acid amplification tests (NAATs) developed, with recent examples applied for the detection of *Fasciola* spp. from whole worm extract, species or strain differentiation, egg detection, and environmental DNA studies (Calvani et al., 2017; Le et al., 2012; Rathinasamy et al., 2018). These methods offer high sensitivity and specificity but are limited being for PCR-based, and therefore requiring extensive nucleic acid extraction protocols and specialised equipment (Lee, 2017). As Fascioliasis is typically prevalent in rural and under-resourced areas, PCR testing is not financially or logistically viable. As such, there is a need for rapid molecular tests, for which loop-mediated isothermal amplification (LAMP) could be a suitable alternative.

Loop-mediated isothermal amplification (LAMP) is well-suited for rapid DNA diagnostics due to amplification carried out using a strand-displacing DNA polymerase active at constant temperatures between 60–65 °C and four-six primers for DNA amplification within 1 h (Notomi et al., 2000). Multiple LAMP assays for pathogen detection have been developed for use in low-resource settings, and in-field use in the absence of a central processing laboratory on a range of pathogens (Amoah et al., 2017; Nzelu et al., 2014; Poole et al., 2017; Tomlinson, Barker & Boonham, 2007). LAMP detection of *Fasciola* spp. have been developed, however they require extensive DNA extraction methods and specialised laboratory settings. These assays are mostly restricted to either whole worm or faecal sampling, requiring a minimum of 2-h for DNA extraction, and a minimum additional hour for LAMP cycling (Ai et al., 2010b; Martínez-Valladares & Rojo-Vázquez, 2016). The total time-to-result, inclusive of DNA preparation and amplification visualisation for current *F. hepatica* LAMP detection is at least 3 h and is reliant on access to a sufficiently equipped central processing laboratory.

Here we report the development of DNA extraction methods and a specific LAMP assay, suitable for field-use that allows rapid, reliable, and robust detection of *F. hepatica* in water and faecal samples within a 1 h. This highlights the potential of the assay to inform local treatment decisions in real-time for management of liver fluke infections.

## Materials and Methods

### *Fasciola hepatica* genomic DNA extraction

Adult *F. hepatica* were sourced from Victorian abattoirs. Briefly, cattle and/or sheep livers were collected and cut into ~10 mm sections following Reichel (2002) with minor modifications: livers were palpated to remove adult flukes from the liver sections, flukes were then washed three times in sterile phosphate buffered saline (100 mM Na_2_HPO_4_, 20 mM KH_2_PO_4_, 1.37 M NaCl, 30 mM KCl, pH 7.4), placed into nucleic acid preservation buffer containing 19 mM EDTA, 18 mM Na_3_C_6_H_5_O_7_, 3.8 M (NH_4_)_2_SO_4_, pH 5.2 (Camacho-Sanchez et al., 2013) and frozen at −20 °C until required for either genomic DNA (gDNA) extraction or egg collection. Genomic DNA was prepared following manufacturer instructions using a Bioneer AccuPrep® Genomic DNA Extraction Kit (Bioneer, Daejeon, South Korea) with minor modifications as follows: 20–25 mg of tissue was cut from the posterior end, minced using a scalpel blade on a petri dish and transferred to a microfuge tube containing tissue lysis buffer pre-heated to 60 °C for further homogenisation with a pellet pestle (Merck, Darmstadt, Germany). Elution was performed in 100 µL volumes using TE buffer (10 mM Tris HCl, 0.1 mM EDTA, pH 8.4) pre-heated to 65 °C and eluted twice to improve recovery. Eluted DNA was assessed using a NanoDrop™ 2000 Spectrophotometer (Thermo Fisher Scientific, Waltham, MA, USA), with absorbance readings for 280/260 and 260/230 ratios between 1.8–2.0 considered acceptable. Samples were stored at −20 °C until required.

### Helminth genomic DNA extraction for specificity panel

Common livestock helminths (Table 1), kindly provided by Dr Tim Elliot (Invetus, Armidale, NSW, Australia), were washed three times with 70% EtOH. Adult helminths were weighed, and 20–25 mg of tissue excised from the anterior end where possible, minced using scalpel blades, and transferred to microfuge tubes. Larvae provided in absolute EtOH were sedimented by centrifugation at 13,000*g* for 15 min and assessed for visible pellet formation. Centrifugation was repeated if no pellet was observed and ethanol was then removed. Total gDNA was extracted following Brindley et al. (1989) with minor modifications as follows: 25 µL grinding buffer containing 80% (v/v) homogenisation buffer (100 mM NaCl, 200 mM sucrose, 10 mM EDTA, 30 mM Tris-HCl pH 8.0) and 20% (v/v) lysis buffer (250 mM EDTA, pH 8.0, 2.5% (w/v) SDS, 500 mM Tris-HCl, pH 9.2), pre-heated to 60 °C was added to each tube containing each helminth species and were mechanically disrupted using pellet pestles (Merck, Kenilworth, NJ, USA). Following incubation at 65 °C for 30 min, 14 µL of 8 M ammonium acetate was added to achieve a final concentration of 1 M, then placed on ice for 30 min. Tubes were centrifuged, 13,000g at 4 °C, for 10 min to pellet precipitated proteins and the supernatant containing DNA was transferred to a fresh microfuge tube for ethanol precipitation.

**Table 1:** Common livestock helminths and life stages used for DNA extraction for specificity panel.

Species	Abbreviation	Life stage
*Teladorsagia circumcincta*	Tec	Adult
*Haemonchus contortus*	Hc	Adult
*Trichostrongylus colubriformis*	Tc	L3
*T. axei*	Ta	L3
*Ostertagia* spp.	Ost	L3
*Cooperia* spp.	Coop	L3
*Paramphistomum cervi*	Pc	L3

Briefly, 2.5 volumes of chilled absolute ethanol and 1:10 ratio 3 M sodium acetate was added to the solution. Samples were inverted and incubated at −20 °C for 60 min, or overnight and pelleted by centrifugation, 13,000g at 4 °C, for 15 min. Samples were checked for visible pellets before removing the ethanol. Pellets were subsequently washed with chilled 70% ethanol, re-pelleted by centrifugation, ethanol removed, and the process repeated at least three times. After the final wash, pellets were inverted and air dried before eluting in 50 µL TE buffer pre-heated to 65 °C. Sample quality and concentration was assessed using a NanoDrop™ 2000 Spectrophotometer as previously described, standardised to a final concentration of 5 ng/µL in TE buffer and stored at −20 °C.

### Plasmid DNA preparation for specificity panel

Recombinant plasmids were needed, owing to the difficulty in obtaining snail individuals of species which either serve as intermediate host, or share the same habitat as *F. hepatica* transmitting snails in South-East Australia. Such synthetic DNA constructs containing the internal transcribed spacer (ITS-2) (Table 2) were cloned into pBHA vectors by Bioneer and transformed into chemically competent XL1 Blue *E. coli* cells (New England BioLabs, Ipswich, MA, USA) following manufacturer instructions. Total plasmid DNA was extracted using the AccuPrep^®^ Plasmid Mini Extraction Kit (Bioneer, Oakland, CA) as per manufacturer instructions, eluting twice with TE buffer pre-heated to 65 °C. Samples were assessed for quality and quantity using a NanoDrop™, standardised to 5 ng/µL, and stored at −20 °C until needed.

**Table 2:** Commonly found Australian snails, key liver fluke transmitting snails, their relevance, and GenBank accession numbers encoding the internal transcribed spacer two region used for plasmid DNA extraction for specificity panel.

Species	Abbreviation	GenBank accession	Relevance
*Physa acuta*	Pa	KF316328.1	Common in South-East Australia, non-fluke transmitting
*Pseudosuccinea columella*	Psc	HQ283261.1	Common in NSW, northern VIC, fluke transmitting
*Galba truncatula*	Gt	KT280457.1	Not in Australia, fluke transmitting
*Austropeplea lessoni*	Al	EU556308.1	Common in Southern and Eastern Australia, non-fluke transmitting
*A. viridis*	Av	EU556313.1	Common in central NSW, southern QLD, fluke transmitting
*A. tomentosa*	At	EU556270.1	Common in South-East Australia, key fluke transmitting species in VIC

### *Fasciola hepatica* quantitative PCR

Quantitative PCR was used to validate and compare initial *F. hepatica* LAMP (FhLAMP) results. A specific quantitative PCR (qFH) assay for *F. hepatica* detection was modified from Rathinasamy et al. (2018) and performed as a singleplex. Reactions were run in 25 µL volumes containing 1× SensiMix II Probe mastermix (Bioline, Alexandra, NSW, Australia), 0.3 µM each of forward and reverse primers, 0.1 µM probe (Table 3), 1 mM MgCl_2_ to achieve a final concentration of 4 mM MgCl_2_, and 2.5 µL template (diluted 1/50 for faecal samples). Amplification was performed using a MIC qPCR cycler (BioMolecular Systems, Upper Coomera, Qld, Australia) under the following cycling conditions: 10 min at 90 °C, followed by 40 cycles of 10 s at 95 °C and 20 s at 60 °C. All PCR runs contained a positive control to monitor intra-assay variation, and a no template control (NTC) containing nuclease-free water (NFW) to monitor contamination. All qPCR runs were assessed using the MIC PCR program (V2.6.4) applying a dynamic normalisation method, with a set threshold of 0.5 normalised fluorescence units, excluding the first give amplification cycles.

**Table 3:** Individual primer sequences used for *Fasciola hepatica* qPCR.

Primer name	Primer direction	Primer sequence 5′–3′
qFhITS-2_FP	Forward	GGTTGGTACTCAGTTGTCA
qFhITS-2_RP	Reverse	CAAAGTGACAGTGACGGAA
qFhITS-2_P	Probe	FAMCCTAGTCGGCACACTTATGATTTCTG-BHQ**-**1

### Further optimisation of a *Fasciola hepatica* LAMP

*F. hepatica* LAMP (FhLAMP) primers (Table 4) were sourced from Martínez-Valladares & Rojo-Vázquez (2016), and two loop primers, LF and LB (Table 4) were manually designed using CLC Genomics (Qiagen, Hilden, Germany) following loop primer design requirements (Nagamine, Hase & Notomi, 2002) Amplification was optimised in final volumes of 25 µL consisting of 15 µL isothermal mastermix (ISO-DR004; OptiGene, Horsham, United Kingdom), 5 µL primer mix with optimised final concentrations of 2 µM inner primers (FIP & BIP), 0.2 µM outer primers (F3 & B3), 2 µM loop primers (LF & LB), and 5 µL template following manufacturer instructions. All reactions contained a positive control to monitor inter-assay variation, and NTC reactions containing NFW to monitor contamination. Reactions were performed in either a Genie II or Genie III fluorometer (OptiGene) with an initial pre-heat of 40 °C for 1 min, followed by amplification at 65 °C for 30 min, and anneal from 94–84 °C at 0.5 °C/s. Results were reported as time to positive (T_p_) in minutes and seconds (mm.ss), with anneal derivative melting temperature (T_a_) reported in degrees Celsius (°C). Performance was determined by assessing the analytical sensitivity and specificity, with inter-assay variation of FhLAMP assessed by performing 10 replicate runs using a 10-fold serial dilution of *F. hepatica* gDNA with starting concentrations of 5 × 10^0^ ng/µL − 5 × 10^−5^ ng/µL in duplicate replicates. The limit of detection was determined where at least 95% of samples recorded T_p_ values with standard deviations below a value of ten, and analytical specificity assessed using prepared helminth and snail DNA as described earlier.

**Table 4:** Individual primer sequences used for *Fasciola hepatica* LAMP.

Primer	Primer sequence (5′–3′)	Sequence region (5′–3′)	Source
FIP	TCTGCCAAGACAAGGGTGCATGTGAGGTGCCAGATCTATGG	F1, F2	Martínez-Valladares & Rojo-Vázquez (2016)
BIP	GTGCAGTGGCGGAATCGTGGTGTGCCGACTAGGGGATC	B1, B2	
F3	GCTGGCGTGATCTCCTCTA	F3	
B3	AACGTGCCTGGTATGGAATT	B3	
LF	ATACATTAGGGAAACGC	LF	This study
LB	AACGTGCCTGGTATGGAATT	LB	

### Determining optimal dilution factor for FhLAMP amplification from cattle faecal samples

Known negative cattle faecal samples confirmed by liver fluke faecal egg counts (LFEC) and coproantigen ELISA (cELISA) used in this study were kindly provided by Dr Jane Kelley, La Trobe University. During initial validation to mimic sample preparation under field conditions and to assess the effects of inhibitory substances on DNA amplification, samples were measured using disposable 5 µL inoculating loops. Two large loopfuls of faeces was transferred into a microfuge tube ranging between 190–350 mg (average 239 mg) faeces per sample. Faecal samples were spiked with decreasing quantities of *F. hepatica* gDNA; 1, 0.5, 0.25, and 0.125 µg equivalent to 1.96 × 10^0^, 9.9 × 10^−1^, 4.98 × 10^−1^, and 2.49 × 10^−1^ ng/µL respectively when back calculated on the assumption of 500 µL faecal content volume. DNA extraction was performed following the method by Matsui et al. (2007) with minor modifications as follows: 500 µL 10% w/v Chelex resin (Bio-Rad, Hercules, CA, USA) prepared in NFW and 50 µL 10% w/v polyvinylpolypyrrolidone (average molecular weight 360,000) was added to samples described above then shaken by hand to form an even homogenate before boiling at 100 °C for 10 min. After boiling, samples were cooled to ambient temperature and to sediment any debris, including the Chelex resin. The supernatant was used for DNA amplification and assessed using a range of dilution factors from neat, 1/10, 1/20, 1/50, 1/100, 1/200, 1/500, and 1/1,000 for each sample to determine the optimal dilution factor required for diluting out faecal inhibitory substances known to interfere with DNA polymerases.

### Acquisition of *F. hepatica* eggs for faecal spiking trials from adults

Eggs were collected from previously obtained adult liver flukes obtained (as described earlier), by placing a single adult in a 35 mm diameter petri dish under a stereo microscope with the specimen placed ventral side up to locate the ventral sucker. The specimen was then covered in MilliQ water to prevent desiccation. Fine forceps and an angled teasing needle were used to gently open the specimen immediately underneath the ventral sucker where the uterus, containing eggs was located and agitated to release eggs into solution. Specimens were then rinsed with MilliQ water using a wash bottle prior to removal to dislodge residual eggs. The contents of the dish were thoroughly washed with MilliQ water into a Falcon® 40 µm cell strainer (Corning, Corning, NY, USA) to ensure no residual eggs remained on the petri dish. The strainer was rinsed three times with MilliQ water before carefully being placed upside down over a 50 mL conical tube and washed with MilliQ water to dislodge any trapped eggs into the tube. Washed eggs were sedimented by centrifugation at 3,000*g* for 10 min and checked for pellet formation before transferring them into a microfuge tube containing 200 µL NFW, covered with foil and stored at 4 °C for up to 1 month, or until required. Prior to use, the egg solution was gently swirled and 50 µL of egg suspension transferred into a 35 mm diameter petri dish to count the required number of *F. hepatica* eggs. Eggs were checked for integrity, discarding damaged eggs, counted and then transferred by pipetting into known negative faecal samples for spiking trials.

### Optimisation of a minimal processing DNA extraction method from cattle faecal samples

Different lysis buffers were trialled to assess which one yielded the most consistent amplification and lowest T_p_ values using minimal processing steps. Each lysis buffer was trialled using known *F. hepatica* negative cattle faecal samples and *F. hepatica* eggs (as described earlier), with lysis buffers assessed in this study described in Table 5 below. For each buffer analysed microfuge tubes containing faecal samples weighing between 190–350 mg (prepared as described earlier) were spiked with 1, 5, 10, 20, and 50 *F. hepatica* eggs each (as described earlier). Samples were vigorously shaken by hand to form an even homogenate before boiling at 95–100 °C for 10 min, then cooled to ambient temperature and allowed to sediment prior to LAMP amplification.

**Table 5:** Lysis buffers used to assess optimal *F. hepatica* egg DNA extraction methods using minimal processing steps.

Buffer name	Composition	Source
Chelex	10% w/v Chelex-resin prepared in nuclease free water, 1:10 vol 10% w/v polyvinylpolypyrrolidone (average molecular weight 360,000)	Adapted from Matsui et al. (2007)
Gram positive lysis buffer (GP)	20 mM Tris-HCl, 2 mM EDTA, 1.2% v/v Triton x-100 Note: lysozyme was omitted	Qiagen DNEasy Blood & Tissue Handbook
Tissue lysis buffer (TL)	100 mM Tris-HCl, 5 mM EDTA, 200 mM NaCl, 0.2% w/v SDS	Adapted from van der Giessen et al. (1999)
SET buffer	10 mM Tris-HCl, 0.5 mM EDTA, 0.2% w/v SDS, 5% w/v Chelex-100	Berry et al. (2007)
Extraction buffer (EB)	100 mM Tris-HCl, 150 mM NaCl, 1% w/v Sarkosyl Note: 5 mM DTT was omitted	Greco et al. (2014)

### Environmental DNA concentration of water samples

Water known to be free from *F. hepatica* was collected from the La Trobe University moat (GPS coordinates: −37.718, 145.044) and spiked with *F. hepatica* gDNA to evaluate a field-appropriate sampling method of circulating free gDNA from water sources. It was necessary to validate the water sampling method this way due to COVID-19 travel restrictions limiting access to *F. hepatica* positive properties. Samples were aliquoted into 50 mL conical tubes and spiked with the same quantities of *F. hepatica* gDNA used for the faecal spiking trials, with starting DNA quantities of 1, 0.5, 0.25, and 0.125 µg added to each aliquot to final concentrations of 2 × 10^−2^_,_ 1 × 10^−2^_,_ 5 × 10^−3^ and 2.5 × 10^−3^ ng/µL per spiked sample. Additionally, 250 mL volumes with the same starting quantities of *F. hepatica* DNA were used (1. 0.5, 0.25, and 0.125 µg), to yield approximate final concentrations of 4 × 10^−3^, 2 × 10^−3^, 1 × 10^−3^, and 5 × 10^−4^ ng/µL each. A negative control of moat water with no spiked gDNA was also included to assess non-target gDNA amplification. Each sample was aspirated through a 50 mL Luer-Lok® syringe before attaching a 0.22 µm Millipore Express (PES) cartridge (Cat #: SVGP1050, Sigma). Contents were filtered until either the entire contents were filtered or the filter was clogged and the filtrate discarded. The syringe and filter were then reversed and the filter contents back drawn, with the resulting eluate transferred to a fresh microfuge tube. A dilution factor of 1/10 was chosen from previous studies for DNA amplification to reduce the presence of DNA amplification inhibitors present from environmental samples (Rathinasamy et al., 2018).

## Results

### Analytical performance of the *Fasciola hepatica* LAMP (FhLAMP)

The analytical performance of FhLAMP was assessed using a ten-fold serial dilution of *F. hepatica* gDNA with starting concentrations ranging from 5 to 5 × 10^−5^ ng/µL. Reactions were performed in duplicate and repeated 10 times to assess analytical sensitivity and inter-assay variation (Table 6). Amplification times ranged from 07.15–22.25 (mm.ss), with T_a_ values ranging from 88.3–89.4 °C. At a starting concentration of 5 × 10^−5^ ng/µL, this resulted in some T_p_ values exceeding 20 min with high inter-assay variation assessed by percent coefficient of variation (CV%) as being above the 10% cut-off value, where amplification was deemed unacceptable due to high T_p_ variability. At this concentration (5 × 10^−5^ ng/µL) amplification was unreliable, with T_p_ values ranging from 16.30–22.30. Therefore 5 × 10^−4^ ng/µL was considered the assay limit of detection producing an average T_p_ value of 13.78, with all replicates consistently amplifying through all ten runs and low inter-assay variation of <5% CV.

**Table 6:** Inter-assay co-efficient of variation of *F. hepatica* gDNA serial dilutions using LAMP.

Starting DNA concentration (ng/µL)	Average T_p_ (mm:ss)	Inter-assay CV (%)
5 × 10^0^	7:32 ± 0.16 SD	2.22
5 × 10^−1^	8:37 ± 0.19 SD	2.28
5 × 10^−2^	9:46 ± 0.31 SD	3.33
5 × 10^−3^	11:05 ± 0.42 SD	3.82
5 × 10^−4^	13:78 ± 0.53 SD	3.88
5 × 10^−5^	19:49 ± 2.48 SD	12.71

**Figure 1:**
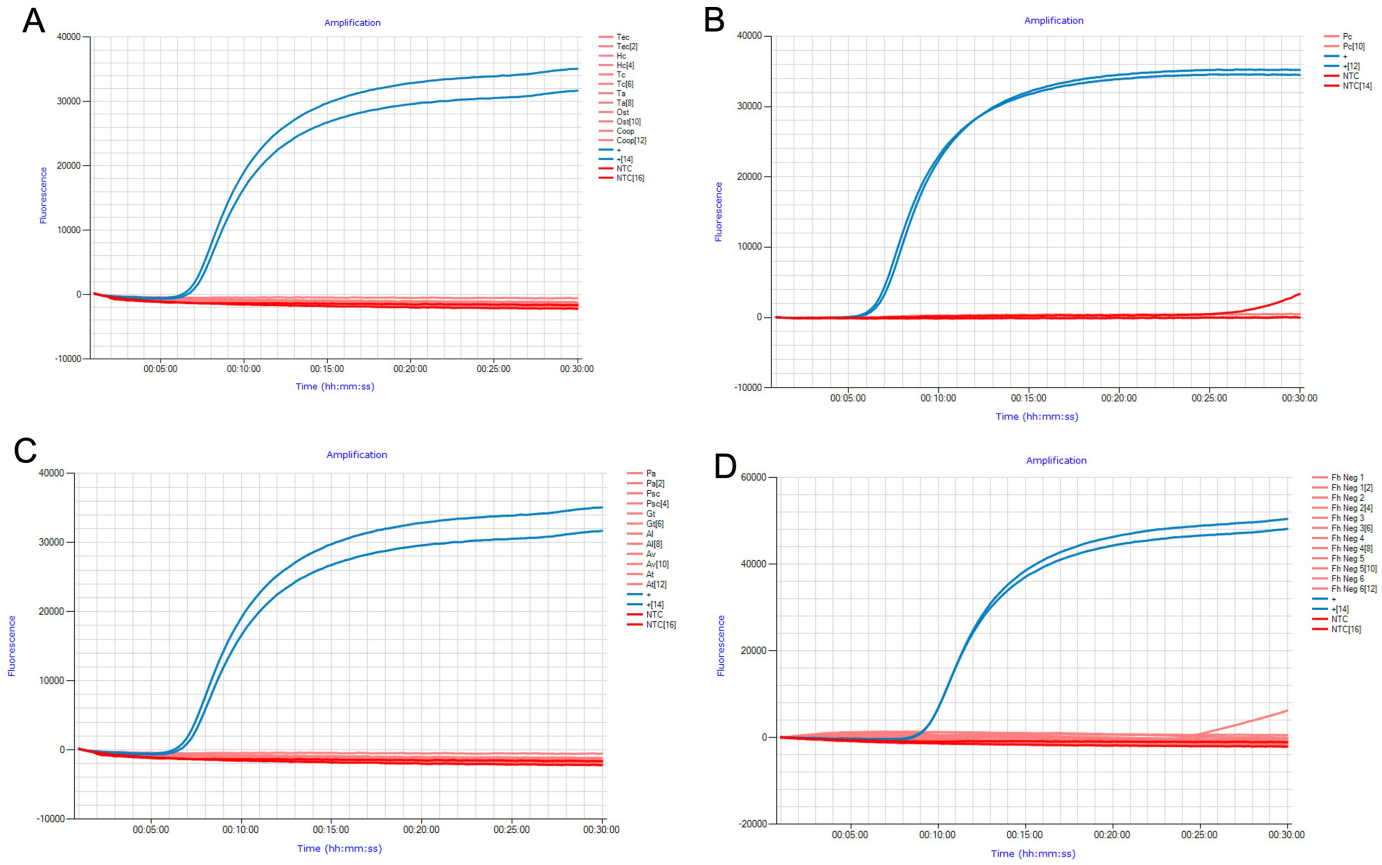
Specificity panel used to determine FhLAMP specificity using common livestock helminths and snail species. (A, B) Specificity panels for FhLAMP using commonly found helminths; (C) snails important in *F. hepatica* ecology and/or lifecycle; (D) assessed non-target amplification from *F. hepatica* free cattle faecal samples. No T_p_ values were observed for all non-target specimens used.

The analytical specificity of FhLAMP was assessed using two measures: (i) gDNA from common livestock helminths (Figs. 1A and 1B) (Table 1) and synthetic constructs of snails (Fig. 1C) relevant to either the *F. hepatica* lifecycle or fluke ecology (Table 2), (ii) assessing non-target amplification from known *F. hepatica* negative cattle faecal samples (Fig. 1D), with all specificity panel results displayed in Fig. 1. Samples were considered negative for *F. hepatica* if no T_p_ values were recorded. Some intermittent ramping was visible in some of the Genie amplification curves after 25 min (Figs. 1B and 1D), however no time to positive detection was recorded for those samples when analysed using the Genie Explorer program or directly on the Genie fluorometer. This is likely due to fluorescence values from these samples failing to exceed the automatically defined detection threshold set by the manufacturer, and were subsequently considered negative. This may be explained by intermittent non-target amplification being occasionally observed in late-stage amplification, probably caused by primer-dimer formation and/or a deterioration in polymerase activity.

No T_p_ values were recorded for all non-target samples used in this study, suggesting that this assay has high specificity even from samples containing large quantities of non-target DNA from a broad range of samples.

### Determining the optimum dilution factor from faecal DNA sampling

**Figure 2:**
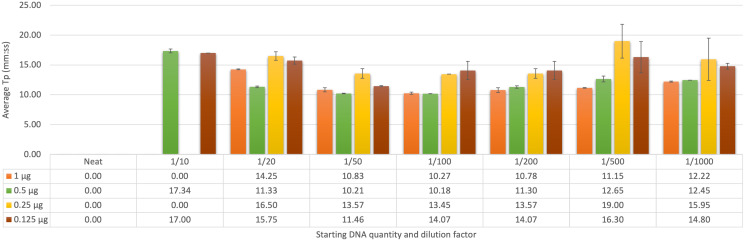
Average time-to-positive (*Tp, represented in minutes:seconds*) values of cattle faecal samples spiked with varying quantities of *F. hepatica* gDNA by LAMP. Multiple dilutions (1/20, 1/50, 1/100, and 1/200) of *Fasciola hepatica* genomic DNA spiked in faecal samples were detected by FhLAMP. Dilution factors producing the lowest T_p_’s were assessed with a 1/50 dilution factor chosen producing the lowest and most consistent T_p_ values. Error bars represent standard deviation of T_p_’s. ^†^ denotes *n* = 1/2 replicates amplifying, hence no standard deviation values.

The initial trial of known negative samples spiked with *F. hepatica* gDNA was assessed using both qFh and FhLAMP, with samples screened using FhLAMP across a range of dilutions from 1 to 1/1,000 (Fig. 2). A dilution factor of 1/50 was chosen as this dilution provided the most reliable T_p_ values ranging from 10–14 min (± ≤0.81 SD) across all starting DNA quantities (Fig. 2C). Although the fastest T_p_ values (between 10–14 min, ± ≤1.52 SD) were observed using a 1/100 dilution (Fig 2D), at lower starting quantities of DNA (from 0.25 µg) amplification was inconsistent or T_p_ values between replicates were highly variable. Dilution factors of 1/20 (Fig. 2B) and 1/200 (Fig. 2E) produced slower T_p_ values (between 11–17 min for 1/20, and 11–15 min 1/200) relative to the 1/50 and 1/100 dilutions. Higher standard deviations and mean T_p_ values of 15.75, ±0.60 SD and 16.30, ±1.52 SD for 1/20 and 1/200 respectively where also recorded at a starting DNA quantity of 0.125 µg in comparison to the 1/50 dilution factor (Fig. 2). This suggests that the amplification at these dilutions may have been affected by either amplification inhibition (1/20) or excess dilution of samples (1/200). All samples prepared using a 1/50 dilution were further analysed using qFh to assess Cq variation (SI 1) between intra-assay replicates to confirm that a 1/50 dilution factor is sufficient for both assays, as faecal samples are known to contain inhibitors that interfere with DNA polymerases. Both qFh and FhLAMP were able to reliably detect *F. hepatica* gDNA from spiked cattle faecal samples with minimal variation between replicates (± ≤0.5 SD qFh, ± ≤0.81 SD FhLAMP), using a 1/50 dilution factor with Cq values for qFh ranging from 26–28, and T_p_ values for FhLAMP from 10–14 min (Table 7). This suggests that a dilution of 1/50 is sufficient for DNA amplification and that subsequent clean-up steps are not necessary prior to DNA amplification.

**Table 7:** Comparison of qFh and FhLAMP amplification values and their standard deviations assessed using spike faecal samples to determine faecal inhibition on *F. hepatica* DNA amplification.

Fh gDNA starting DNA quantity diluted 1/50	qFh average Cq	FhLAMP average T_p_
1 µg	26.59 ± 0.30 SD	10:83 ± 0.34 SD
0.5 µg	26.15 ± 0.13 SD	10:22 ± 0.05 SD
0.25 µg	28.84 ± 0.03 SD	13:58 ± 0.81 SD
0.125 µg	28.61 ± 0.05 SD	11:47 ± 0.09 SD

### Comparison of lysis buffers for use in cattle faecal samples spiked with *F. hepatica* eggs

**Figure 3:**
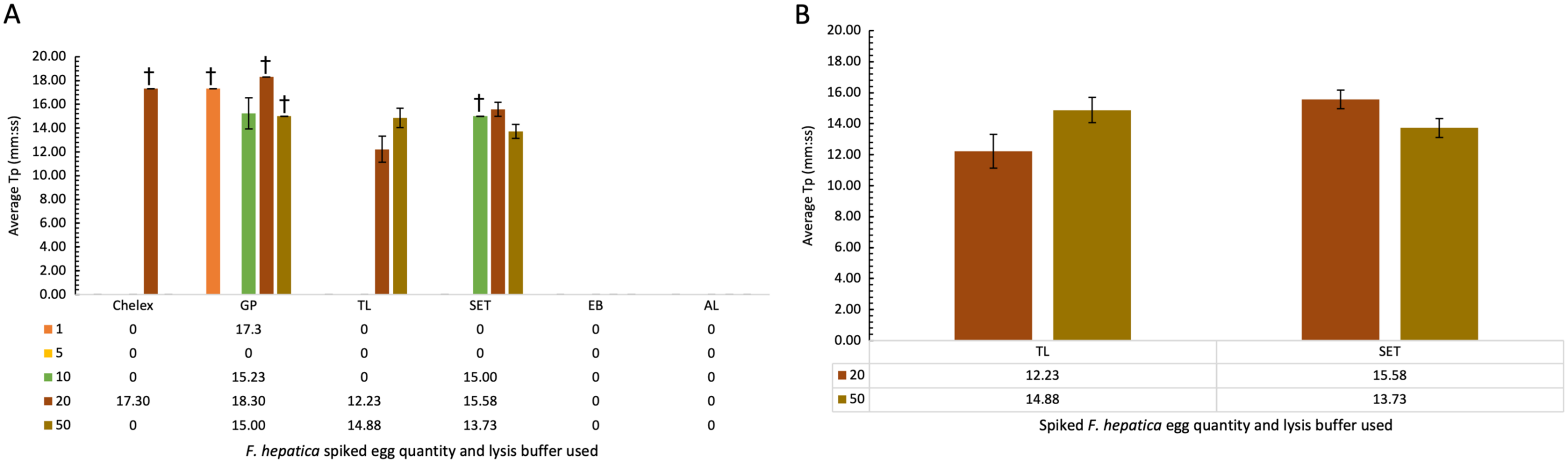
(A–B) Average time-to-positive (*Tp, represented in minutes:seconds*) values of cattle faecal samples spiked with varying quantities of *F. hepatica* eggs, detected by FhLAMP. Average Tp values and standard deviations of *F. hepatica* negative cattle faecal samples spiked with varying quantities of *F. hepatica* eggs, assessed using different lysis buffers for use in FhLAMP using minimal processing methods for DNA extraction. Samples were diluted 1/50 and the most consistent T_p_ values for each lysis buffer assessed, with SET buffer chosen producing the most consistent amplification with lowest T_p_ standard deviation relative to other lysis buffers used. Error bars represent standard deviation of T_p_ values. ^†^denotes *n* = 1/2 replicates amplifying, hence no standard deviation values.

Cattle faecal samples were spiked with known quantities of *F. hepatica* eggs containing: 1, 5, 10, 20, and 50 eggs per lysis buffer. Samples were boiled for 10 min at 95–100 °C for DNA extraction using the minimal processing method described earlier and assessed by FhLAMP. Various lysis buffers were used as detailed in Table 5, and a dilution factor of 1/50 chosen for all samples. Figure 3A represents the mean of all duplicate replicate T_p_ values using different lysis buffers, with extraction and alkaline lysis buffers failing to amplify any *F. hepatica* DNA. Additionally, Chelex and gram-positive buffers amplified inconsistently, with one of two duplicate replicates returning T_p_ values. Tissue lysis and SET buffers returned positive T_p_ values for both replicates from samples containing 20 and 50 eggs with an average T_p_ for samples prepared using tissue lysis buffer of 12.22 (±1.10 SD) and 14.88 (±0.81 SD) for 20 and 50 eggs respectively, with mean T_p_ values of 15.58 (±0.60 SD) and 13.73 (±0.60 SD) for 20 and 50 eggs prepared using SET buffer (Fig. 3B). As standard deviations were higher in samples prepared using tissue lysis buffer, SET buffer was therefore determined to be the optimal lysis buffer for use in *F. hepatica* egg DNA extraction from faecal samples, consistently amplifying *F. hepatica* DNA from as little as 20 eggs per sample.

### Evaluation of an extraction free water DNA isolation method

Water known to be *F. hepatica* free was collected from the La Trobe University moat and used to assess an extraction-free DNA capture method suitable for field-use and LAMP detection. Each sample, containing either 50 or 250 mL of water was spiked with decreasing quantities of DNA (1, 0.5, 0.25 and 0.125 µg) and a negative extraction control of untreated moat water, was filtered and diluted 1/10 for use in FhLAMP assays. The average T_p_ values for each sample, with all spiked samples using 50 mL volumes amplifying, returned T_p_ values ranging from 10.30–20.15 (mm.ss) (± ≤4.21 SD) (Table 8). Increasing sample volume to 250 mL, whilst retaining the same starting DNA quantities, provided slower T_p_ values of 13.15–21.15 (mm.ss) (± ≤5.06 SD) and samples spiked with 0.125 µg *F. hepatica* gDNA failed to amplify. This is likely due to the Sterivex® filters clogging after ~100 mL and therefore were unable to filter the entire sample volume to sufficiently concentrate the free DNA in that sample. Regardless, this suggests that the extraction free method of DNA capture described here is suitable for field-use with all 50 mL spiked samples and three of four 250 mL spiked samples recording positive T_p_ values. Despite whole gDNA being spiked into moat water, samples containing high levels of competing non-target DNA and DNA amplification inhibitors with no DNA denaturation or clean-up steps used, produced detection levels as low as 1 × 10^−3^ ng/µL *F. hepatica* gDNA from partially filtered 250 mL spiked water amplifying in under 20 min.

**Table 8:** Known negative *F. hepatica* free water spiked with varying starting quantities of *F. hepatica* gDNA, used to assess minimal DNA sample preparation methods from 50 and 250 mL water samples.

	50 mL volume	250 mL volume
DNA starting quantity (µg)	Average T_p_ (mm:ss)	Average T_p_ (mm:ss)
1	12:98 ± 2.11 SD	15:30 ± 3.04 SD
0.5	13:80 ± 1.80 SD	17:58 ± 5.06 SD
0.25	13:73 ± 1.75 SD	17:00 ± 1.41 SD
0.125	14:23 ± 4.21 SD	ND
Neg	ND	ND

**Note:**

ND = Not detected.

## Discussion

Fascioliasis is an economically important, zoonotic disease affecting livestock. Infection heavily reduces animal production yields, with additional costs required for treatment and management of this disease (Young et al., 2011). Infections are further compounded with the increasing prevalence of TCBZ-R flukes globally as there are limited alternative flukicides (Walker et al., 2004). Until a long-term treatment such as a vaccine is available, additional methods to manage fluke abundance should be considered. An option is implementing integrated parasite management (IPM), which includes a multi-faceted approach to manage liver fluke prevalence and assists in reducing anthelmintic overreliance (Kahn & Woodgate, 2012; Kelley et al., 2016; Woodgate & Besier, 2010). Real-time diagnostics for the detection of *Fasciola* spp. in various environmental samples will improve non-chemical management of *Fasciola* spp. infection.

We have described the development and optimisation of the *Fasciola* LAMP with field-applicable environmental sampling, with decreased amplification times (<20 min down from >1 h), and five-fold increased sensitivity (5 × 10^−4^ ng/µL compared to 1 × 10^−3^ ng/µL) through the addition of loop primers to the previously developed *Fasciola* LAMP assay by Martínez-Valladares & Rojo-Vázquez (2016). Although additional primers could have potentially impacted FhLAMP specificity, no cross-reactivity was observed from non-target helminths particularly *C. daubneyi* and *P. cervi* which are frequently reported co-infections in cattle (Gordon et al., 2013), various snail species, liver fluke free faeces or environmental water samples.

Current LAMP assays for *Fasciola* spp., are reliant on DNA extraction kits requiring well-equipped facilities (Ai et al., 2010b; Arifin, Höglund & Novobilský, 2016; Martínez-Valladares & Rojo-Vázquez, 2016). These studies report amplification times between 55–70 min and detection limits between 1 × 10^–3^ and 1 × 10^−5^ ng/µL, requiring an additional 1 h for DNA preparation. In comparison, the FhLAMP was completed within 1 h, requiring 15 min for crude DNA preparation and amplification completed within 30 min irrespective of starting material used whilst improving sensitivity from previous studies above to 5 × 10^−4^ ng/µL.

Field-appropriate sampling for faeces and water was assessed using the improved FhLAMP as collection of these is non-invasive and provides indicators of fluke presence. Currently no crude methods are available in the literature for *F. hepatica* faecal egg detection which may be attributed to eggs requiring extensive mechanical disruption and multiple clean-ups to remove common PCR inhibitors in faecal contents (Ai et al., 2010a; Calvani et al., 2017). Therefore the crude lysis buffer, boiling and diluting method was used to prepare DNA extracts from faeces containing *F. hepatica* eggs, and SET buffer described in Berry et al. (2007) providing the most consistent T_p_ values with FhLAMP detecting from 20 eggs within 190–350 mg spiked faeces.

These results equate to a sensitivity of approximately 83 eggs per gram (EPG) of stool indicative of high fluke burdens, which is within range of the diagnostic performance for currently used copromicroscopy methods being LFEC and mini-FLOTAC, with sensitivities of 96 EPG or greater (Zárate-Rendón et al., 2019). Although the crude extraction method can detect from 83 EPG compared to FlukeFinder which reports detection from 5 EPG (Reigate et al., 2021), the FhLAMP reduces total hands-on time and enables multiple samples to be processed simultaneously, however for zoonotic cases where sensitivity offered with FlukeFinder is prioritised over throughput, the FhLAMP may miss low-level infections. In contrast for livestock management in herds or flocks where fluke burdens of ≥40 are considered the threshold for economic losses in cattle, the FhLAMP may be suitable to detect high infections and minimise ill-thrift (Vercruysse & Claerebout, 2001). As such, the extraction of DNA from *F. hepatica* eggs in the field warrants further investigation to improve the sensitivity of any field-based nucleic amplification technique.

An additional source of amplifiable DNA from faeces could be cell-free DNA through tegumental sloughing and degraded or non-viable metacercariae and/or eggs as adult flukes reside in host biliary ducts producing eggs that are shed through faeces (Cameron et al., 2017; Wilson et al., 2011). The cumulative presence of cell-free DNA, eggs and metacercariae in infected animals is likely sufficient for the positive detection of *F. hepatica* DNA despite low or no egg counts from *F. hepatica* DNA being shed, supported by Martínez-Valladares & Rojo-Vázquez (2016) reporting positive LAMP detection of *F. hepatica* DNA 1 week post-challenge from faecal samples, before fluke maturation and egg production have occurred.

While our study was unable to assess whether *F. hepatica* free DNA could be detected from fluke positive faecal samples, our results from the faecal gDNA spiking suggest that the FhLAMP presented here can detect free *F. hepatica* DNA from a final concentration of at least 2.49 × 10^−1^ ng/µL *F. hepatica* gDNA in 500 µL unpurified faecal contents further diluted 1/50, with amplification recorded in under 12 min.

A second sampling method using environmental water samples was used for the detection of free DNA shed by the parasite in the environment, referred to as environmental DNA (eDNA). The source of this DNA could be from eggs contained in faeces washed into water sources including shallow puddles known to harbour *F. hepatica* transmitting snails, delvers, or troughs, therefore possibly harbouring the aquatic developmental stages of *F. hepatica* being miracidia, sporocysts, rediae, cercariae, or metacercariae (Young et al., 2010). Though this does not differentiate between life stages, it is still a useful indicator of recent fluke presence in the environment as eDNA is short-lived and rapidly deteriorates, with degradation reported at the time of collection, or within days of species removal from the environment (Renshaw et al., 2015; Wood et al., 2020). Further, conventional eDNA sampling is generally processed using large volumes exceeding 500 mL through a vacuum-manifold pump in addition to the requirement of time-consuming DNA clean-up methods which necessitate a central laboratory for sample processing using either costly spin-column kits, or carcinogenic reagents for phase-separation such phenol:chloroform:isoamyl alcohol for DNA extraction (Djurhuus et al., 2017).

Here we present a simple method of concentrating DNA from environmental water samples suitable for use in FhLAMP, amplifying as little as 1 × 10^−3^ ng/µL *F. hepatica* gDNA back calculated from 250 mL spiked moat water, partially filtered. Though the sensitivity in this study is ten-fold less than the results obtained by qPCR in Rathinasamy et al. (2021), reported as 1 × 10^−4^ ng/µL, filtering greatly reduced processing time to less than 15 min for five water samples irrespective of starting volume (50 or 250 mL), down from at least 1 h for spin-column or eDNA clean-up methods (Czeglédi et al., 2021; Rathinasamy et al., 2021). Despite high levels of non-target DNA and DNA polymerase inhibitors present in freshwater samples, amplification of filtered water samples using FhLAMP maintained high specificity, correctly amplifying all samples containing *F. hepatica* gDNA despite the presence of large invertebrate species known to inhabit freshwater, including freshwater shrimp, mosquito larvae, midges, and nymphs (Gooderham & Tsyrlin, 2002; McNally et al., 2013). Additionally, mean *F. hepatica* eDNA quantity detected over seven sampling periods in Rathinasamy et al. (2021) was 2.4 × 10^−3^ ng/µL (2IQR: 9.9 × 10^−4^ ng/µL). This suggests that the extraction-free method reported here is sufficient for *F. hepatica* eDNA sampling, despite partial volumes of ~100 mL of 250 mL spiked samples filtered, with FhLAMP detecting 1 × 10^−3^ ng/µL using this crude concentration method.

## Conclusions

We have developed a highly specific and sensitive LAMP assay for detection of *F. hepatica* in conjunction with two sampling methods suitable for in-field testing. Faecal sampling enables the identification of host infection status, whereas the water sampling method enables the detection of parasite life stages present in the environment. Both methods used together and in conjunction with FhLAMP are useful for integration into IPM programs aimed to mitigate disease burden and parasite presence on a property, which will in turn reduce the use of anthelmintics. Results obtained using this molecular workflow can be deployed for surveillance of *F. hepatica* prevalence on a farm in infected animals and/or around the property. Further in-field studies of both sampling methods are required for validation; however, such methods provide an alternative to traditional commercial kit extractions, greatly reducing both processing time and cost. This is most beneficial to farming properties and locations where central laboratory access is not feasible and primarily where Fascioliasis is endemic. Additionally, the methodologies presented here could be modified for use in other economically important helminth ruminant parasites, with data generated feeding into IPM approaches to mitigate the economic losses incurred by helminth infection in livestock.

## Supplemental Information

10.7717/peerj.13778/supp-1 Supplemental Information 1 Raw numerical data for qPCR and LAMP assays. The raw data from qPCR and LAMP data shown indiviual on each sheet

## Additional Information and Declarations

### Data Availability

The following information was supplied regarding data availability: The raw data of LAMP and qPCR are available at figshare: Beddoe, Travis (2022): Environmental detection of *Fasciola hepatica* by loop-mediated isothermal amplification. La Trobe. Dataset. https://doi.org/10.26181/18822374.v1.
